# Elastic Correlation Adjusted Regression (ECAR) scores for high dimensional variable importance measuring

**DOI:** 10.1038/s41598-021-02706-0

**Published:** 2021-12-02

**Authors:** Yuan Zhou, Botao Fa, Ting Wei, Jianle Sun, Zhangsheng Yu, Yue Zhang

**Affiliations:** 1 Department of Bioinformatics and Biostatistics, School of Life Sciences and Biotechnology, Shanghai Jiao Tong University, Shanghai, China; 2 Department of Mathematical Sciences, University of Cincinnati, Cincinnati, USA

**Keywords:** Computational biology and bioinformatics, Genetics

## Abstract

Investigation of the genetic basis of traits or clinical outcomes heavily relies on identifying relevant variables in molecular data. However, characteristics such as high dimensionality and complex correlation structures of these data hinder the development of related methods, resulting in the inclusion of false positives and negatives. We developed a variable importance measure method, termed the ECAR scores, that evaluates the importance of variables in the dataset. Based on this score, ranking and selection of variables can be achieved simultaneously. Unlike most current approaches, the ECAR scores aim to rank the influential variables as high as possible while maintaining the grouping property, instead of selecting the ones that are merely predictive. The ECAR scores’ performance is tested and compared to other methods on simulated, semi-synthetic, and real datasets. Results showed that the ECAR scores improve the CAR scores in terms of accuracy of variable selection and high-rank variables’ predictive power. It also outperforms other classic methods such as lasso and stability selection when there is a high degree of correlation among influential variables. As an application, we used the ECAR scores to analyze genes associated with forced expiratory volume in the first second in patients with lung cancer and reported six associated genes.

## Introduction

As a result of novel biotechnology such as next-generation sequencing (NGS) technologies, genomic and clinical research have benefited dramatically from the steep increase in both quantities and quality of molecular data. Identifying important genomic factors correlated with phenotypes or clinical outcomes will help scientists investigate the genetic basis of traits or diseases and make targeted interventions possible. An example is the identification of cancer driver genes that are crucial for diagnosing and treating the disease. On the other hand, the data complexity challenges the analysis methods. In these data, the number of variables is often much larger than the number of individuals. For example, expression of > 20,000 mRNA transcripts can be measured using microarrays^1^, but a few of the experiments have fewer than 100 samples^2^. More importantly, complex correlation patterns^3–5^ and considerable interactions^6^ are present between the variables (e.g., genes, SNPs). Additionally, the number of causal or relevant variables to clinical outcomes may be small^7–9^.

Many computational tools have been developed to help the selection of genomic factors relevant to the quantitative traits or clinical outcomes. The most commonly used approach performs independent hypothesis testing on each variable, and keep those whose $p$ values are below the significance threshold, which unavoidably leads to high rates of false positives. However, adjusting the threshold with multiple comparison criteria, such as Bonferroni or False Discovery Rate (FDR) correction, will cause variables of small to moderate effects to be erroneously discarded^10^, thus introducing many false negatives. Another class of methods are penalized regression models (e.g., lasso^11^, elastic net^12^, minimax concave penalty^13^), which aim to select a small set of predictors that are associated with a trait. Despite their good performance in prediction, they face challenges in association studies. For example, the lasso tends to select only one variable from a group of highly correlated genomic factors, and it cannot select more variables than the sample size. The elastic net addresses these two problems, but its result, like other well-known approaches (lasso, minimax concave penalty), can be numerically unstable when applying cross-validation to estimate the parameters. Some researchers also propose to rank and select variables based on their assigned scores. An example of this is the variable importance computed by random forest^14^, which has been applied in genetics^15^, gene expression^16^, methylation^17^, proteomics^18^, and metabolomics studies^19^. Another example is stability selection^20^, which is based on linear models and has flavors of both lasso and random forests. Comparing with the lasso, it is more suitable for variable selection, but the price to pay is the reduced power to identify the true signals. CAR scores^21^ and CARS scores^22^ also fall into this group, they are easy to calculate, but might not be flexible enough when the noise in data is too small or too large.

Due to these limitations of current approaches, we developed the Elastic Correlation Adjusted Regression (ECAR) score for simultaneously variable selection in high dimensional biological data. This method is an extension of the CAR scores^21^ and improves over the CAR scores in terms of selection accuracy by adjusting the parameter according to different datasets’ characteristics. Specifically, the ECAR scores aim to rank the truly influential variables as high as possible. To determine the final selected set, we apply the adaptive false discovery rate density approach^23^. We compared the ECAR scores’ performance to lasso, stability selection, ridge, CAR scores, and Sure Independence Screening^24^ (SIS) on three classes of datasets: simulated datasets with a fixed correlation structure, semi-synthetic datasets generated from mRNA expression data, and real datasets from T3/barley database. In our study, we showed that ECAR improves CAR and rivals popular methods like the lasso in terms of the variable selection accuracy and the predictive power of high-rank variables.

## Results

### The idea of ECAR scores

Suppose we have random variables (${\varvec{X}}_{p \times 1} ,\user2{ }Y_{1 \times 1} )$, where ${\varvec{X}} = (X_{1} , \ldots ,X_{p} )^{{\varvec{T}}}$ denotes the genomic features and $Y$ is the outcome, ECAR scores ${\varvec{\omega}}$ are defined as

1 $$\begin{array}{*{20}c} {\omega = {\varvec{R}}^{ - \alpha } {\varvec{R}}_{{{\varvec{X}}Y}} ,} \\ \end{array}$$

where ${\varvec{R}}$ is the correlation matrix of the feature space, and ${\varvec{R}}_{{{\varvec{X}}Y}}$ is the Pearson correlation coefficient vector. ${\varvec{R}}^{ - \alpha }$ is the αth power of the real symmetric matrix ${\varvec{R}}$, which is obtained by first computing the spectral decomposition of ${\varvec{R}} = {\varvec{Q}}{{\varvec{\Lambda}}}{\varvec{Q}}^{ - 1}$, and subsequent modification of the resulting eigenvalues ${\varvec{R}}^{ - \alpha } = {\varvec{Q}}{{\varvec{\Lambda}}}^{ - \alpha } {\varvec{Q}}^{ - 1}$. When $\alpha = 0$, the ECAR scores are equivalent to the Pearson correlation coefficients. When $\alpha = 1$, it is equivalent to the semi-partial correlation coefficient. CAR fixes the parameter $\alpha$ at 0.5, and therefore it might be in the middle of the marginal method (${\upalpha } = 0$) and the conditional method (${\upalpha } = 1$).

**Figure 1:**
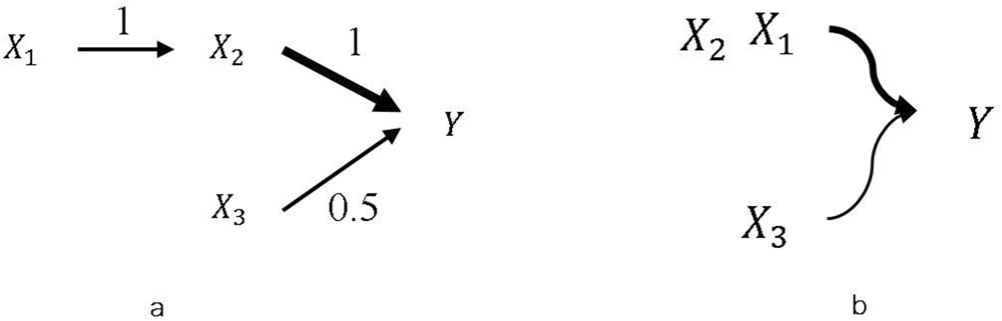
Data generating diagram and univariate contribution of $X_{1}$,${ }X_{2}$,${ }X_{3}$ to $Y$. (**a**) The causal diagram of our data-generating model. (**b**) The univariate contribution of each variable. The overlapped area represents shared explained variance of each variable in univariate regression.

We believe that it is not reasonable to fix $\alpha$ at either 0 (Pearson correlation) or 0.5 (CAR). A small example can illustrate this idea. Suppose $X_{1} \sim N\left( {0,1} \right),{ }X_{2} = X_{1} + \varepsilon , X_{3} \sim N\left( {0,1} \right), Y = X_{2} + 0.5X_{3}$, where $\varepsilon \sim N\left( {0,1} \right)$. Figure 1 shows the diagram and contributions of the three variables. The area of the three circles represent three variables’ univariate contribution to $Y$, which can be seen as the coefficient of determination ($R^{2}$) of 3 univariate regressions. $X_{1}$ circle overlaps with $X_{2}$ circle since $X_{1}$ is not in the data-generating model-its contribution is borrowed from $X_{2}$. Generate 1000 samples from our model and set $\alpha = 0$, the ECAR scores of $X_{1}$, $X_{2}$, $X_{3}$ are 0.56, 0.81 and 0.24 respectively. We noticed that even if $X_{1}$ plays no role in generating $Y$, its score is higher than $X_{3}$, which is in the model. Set $\alpha = 0.5$ and this results in the scores of 0.3, 0.75, and 0.24. $X_{1}$’s score is reduced, however, it is still larger than $X_{3}$’s score. If we set $\alpha = 1$, the three scores will be − 0.03, 0.83, and 0.24, the absolute values of which are much more reasonable in terms of evaluating their actual contributions.

Fixing the $\alpha$ at one is also not ideal. In this case, the scores are equivalent to multiple regression coefficients when ${\varvec{X}}$ and $Y$ are standardized. This seems to be more suitable to locate the truly influential variables, since the non-influential variables will contribute nothing to the outcome given other variables in theory. However, accurate estimation of scores may be difficult as a result of the high degree of correlation among genomic factors^25^. This problem can be solved by shrinkage methods, which means only a limited amount of information from the correlation matrix will be used, and this amount can be seen as parameter $\alpha$ in the ECAR scores.

**Figure 2:**
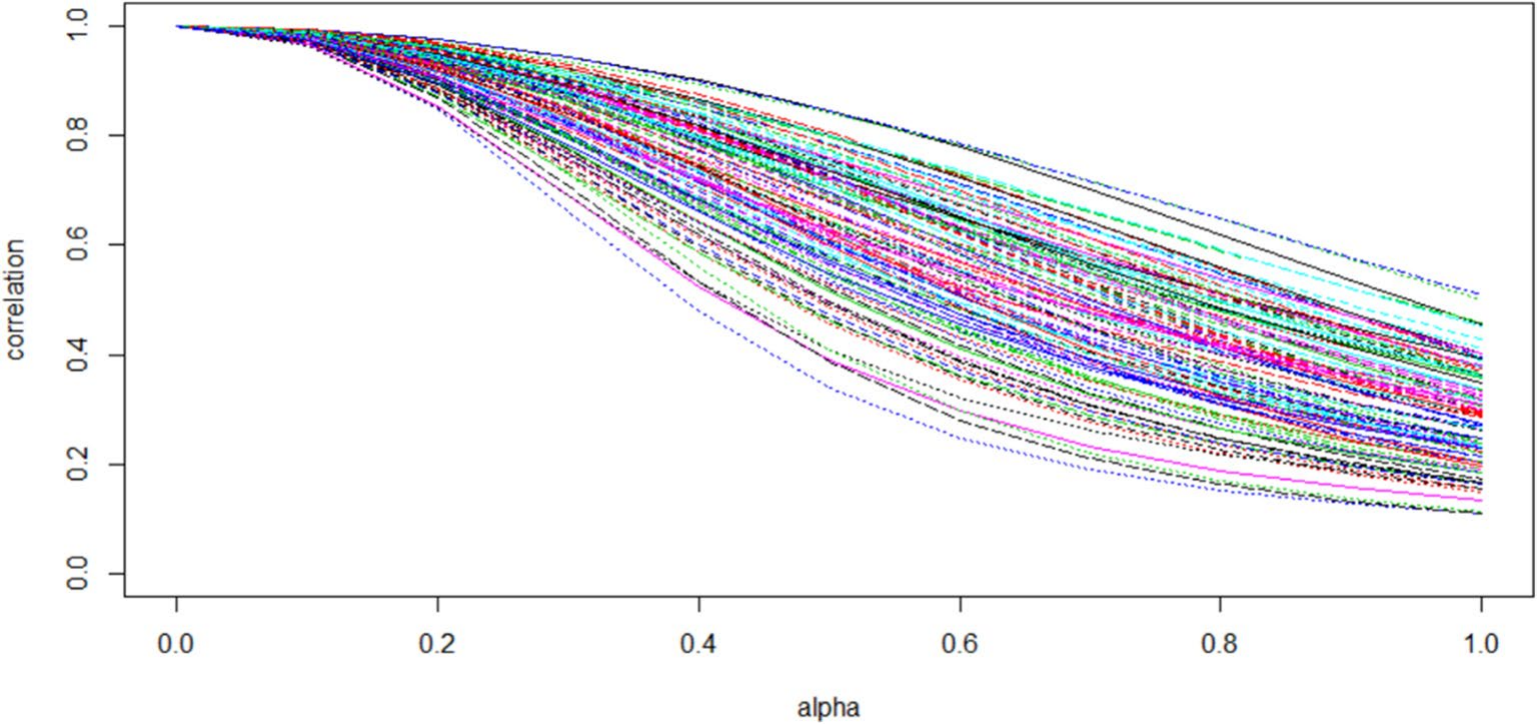
The correlations between 100 transformed gene expression profiles ${\varvec{R}}^{ - \alpha } {\varvec{X}}$ and their original versions ${\varvec{X}}$ as $\alpha$ moves from 0 to 1. Each line of different color represents an mRNA. The features are 100 gene expression profiles selected from The Cancer Genome Atlas (TCGA) LIHC cohort.

$\alpha$ should be adjusted in each dataset to achieve a reasonable extent of compromise between two extremes (multiple regression coefficients and Pearson correlation coefficients). As $\alpha$ varies from 0 to 1, it borrows more and more information from the correlation matrix, and the ECAR scores tend to be more like multiple regression coefficients. The ECAR scores can also be seen as the correlation coefficient between the outcome $Y$ and the transformed features ${\varvec{R}}^{ - \alpha } {\varvec{X}}$. The transformed features ${\varvec{R}}^{ - \alpha } {\varvec{X}}$ will tend to be more “dislike” its original version ${\varvec{X}}$ as $\alpha { }$ increases, and this idea is illustrated in Fig. 2.

Best $\alpha$ moves towards 1 when $R^{2}$ increases, which is a trend consistently observed in our simulations and applications. To estimate $\alpha$ of ECAR scores in different datasets, we proposed a method that can maximize the variable selection power, which is discussed in detail in the Methods section. In brief, first, we need to estimate the $R^{2}$ and the number of influential variables $s$, and then we randomly select $s$ variables and simulate a new dataset using a linear model which has the same ${\varvec{X}}$, $R^{2}$ and $s$ as the real dataset. The $\alpha$ which has the best variable selection performance measured by Area Under Prediction-Recall Curve (PR-AUC) can be found in the new dataset, as truly influential variables are known. This process is performed many times, and we take the median of the best ${\upalpha }$ to be our final estimate, which performs well in general under different correlation structures. In this way, we can predict the $\alpha$ which ranks the influential variables as high as possible in the real problem. This method works well because while we know nothing about the truly influential variables and their correlation structure, we can estimate the parameter by simulating the possible scenarios. The estimated $\alpha$ would remain reasonable in the real setting as long as it is not too far from our simulated ones.

### Comparison of feature selection accuracy on simulated datasets

We compared the performance of ECAR with CAR, SIS, ridge, lasso, stability selection (Details of these methods can be found in Methods section) on 500 simulated datasets consisting of 200 observations and 600 features. The correlation matrix of the features is block diagonal with the compound symmetry structure which was used in the previous research^26^. It is constructed by two equally sized blocks. In each block, the correlation between any two features is 0.25, while variables from different blocks are independent. We used the method described in the Methods section to determine the best $\alpha$. Assume in the linear data-generating model (2) that there are 30 influential variables randomly selected from the first block, and their corresponding coefficients are sampled from the uniform distribution with minimum 0 and maximum 1. $\varepsilon$ is normally distributed with mean 0 and variances $\sigma^{2}$.

2 $$\begin{array}{*{20}c} {Y = X\beta + \varepsilon } \\ \end{array}$$

**Figure 3:**
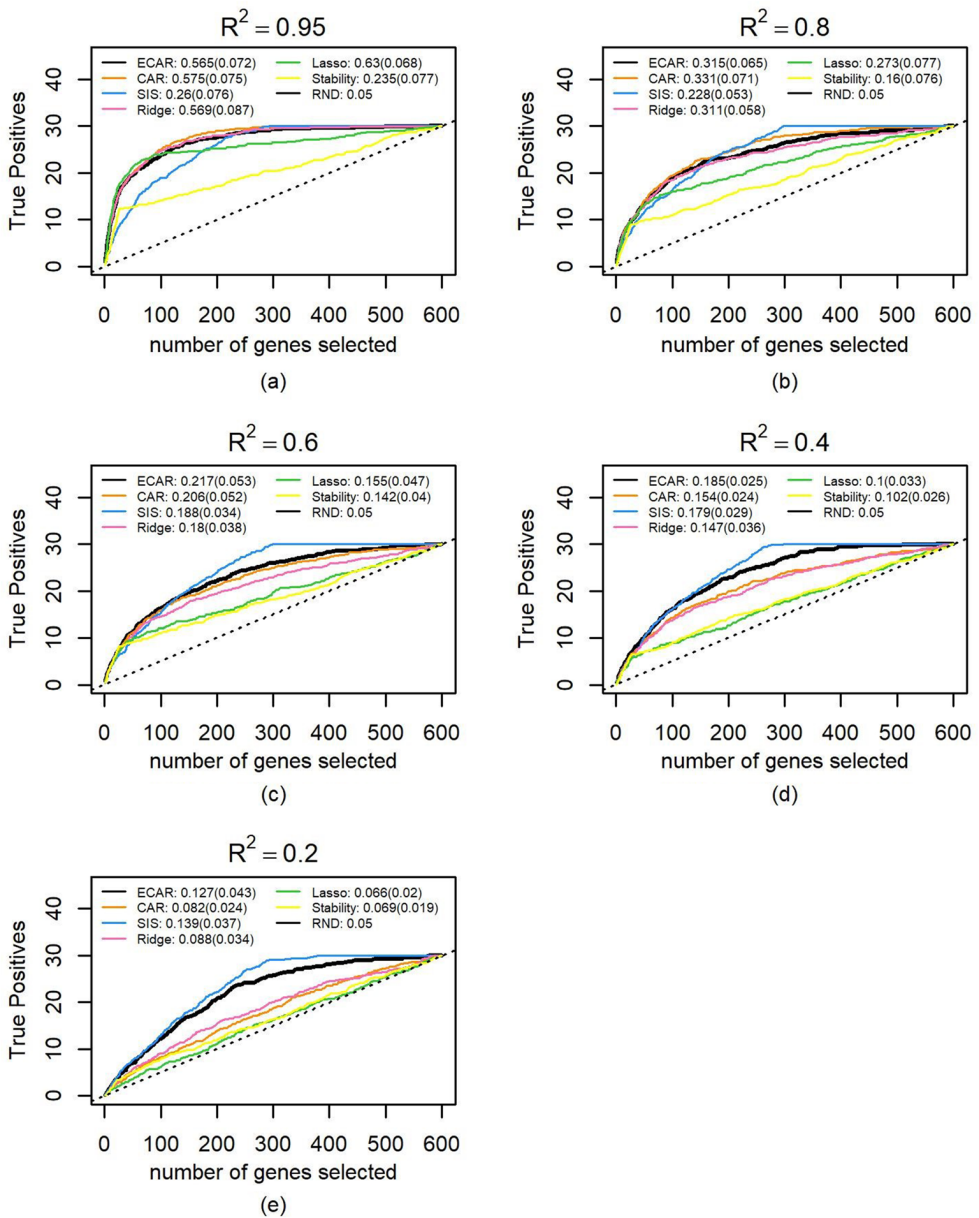
Comparison of feature selection performance on 500 simulated datasets. The median number of true positive variables as a function of the total number of selected genes as well as the median of PR-AUC and its standard deviation are shown for ECAR, CAR, SIS, ridge, lasso and stability selection under five $R^{2}$ scenarios. The total number of influential genes is 30, which are randomly selected from the first 300 genes (first block). Parameter ${\upalpha }$ of ECAR is estimated using the methods described in the Methods section. The regularization parameter of ridge and lasso is estimated using fivefold cross-validation and generalized cross-validation, respectively. As lasso cannot select more variables than the sample size, we let it choose genes randomly when all genes in the output selected set are chosen. (**a**) $R^{2}$ is controlled at 0.95 for the 100 simulated datasets. (**b**) same as (**a**), $R^{2}$ controlled at 0.8. (**c**) Same as a, $R^{2}$ controlled at 0.6. (**d**) same as (**a**), $R^{2}$ controlled at 0.4. (**e**) Same as **a**, $R^{2}$ controlled at 0.2.

**Figure 4:**
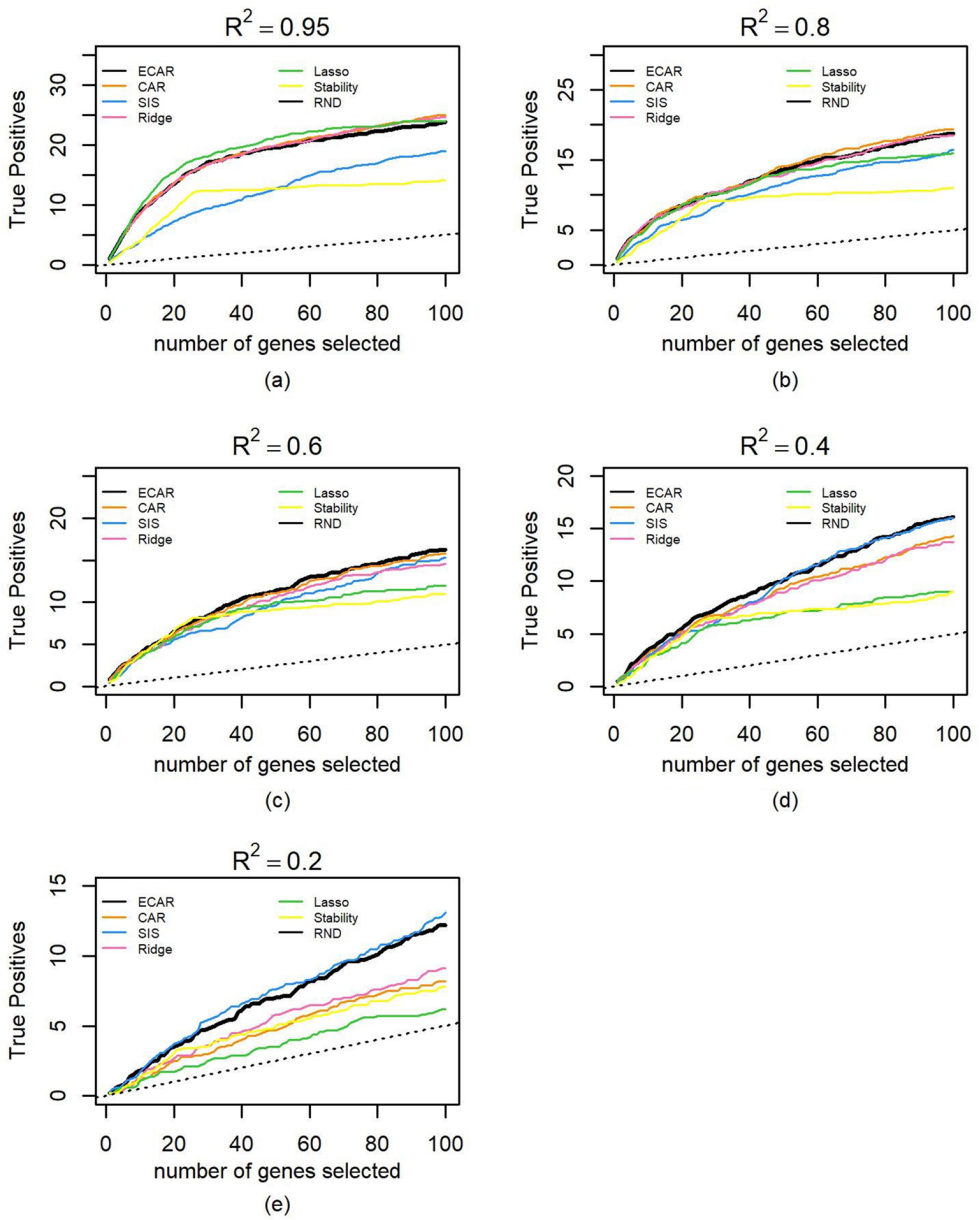
Comparison of feature selection performance on 500 simulated datasets. This figure is the same as Fig. 3 except that paths are truncated at 100 genes.

We adjusted $\sigma$ to synthetic five groups of datasets. Each group consisted of 100 datasets and achieved a different level of the $R^{2}$. Using the methods described in the Methods section to estimate $\alpha$, we noticed that when $R^{2}$ is controlled at 0.2, 0.4, 0.6, 0.8, 0.95, the medians and standard deviations of 100 best $\alpha$ are 0.225 ± 0.22, 0.350 ± 0.17, 0.450 ± 0.13, 0.600 ± 0.10 and 0.750 ± 0.08, respectively. These median values were substituted into the ECAR scores for comparison with other methods on these datasets. The true positives path, as well as the medians and standard deviations of PR-AUC, are shown in Figs. 3 and 4 (a truncated version of Fig. 3) for each method. Under all $R^{2}$ settings, the path of ECAR is among or near the highest paths. The results also demonstrate ECAR’s advantage of flexibility: as the $R^{2}$ drops, SIS outperforms other methods and continues to extend its lead; meanwhile, the $\alpha$ in ECAR decreases, and therefore ECAR behaves more like SIS.

**Figure 5:**
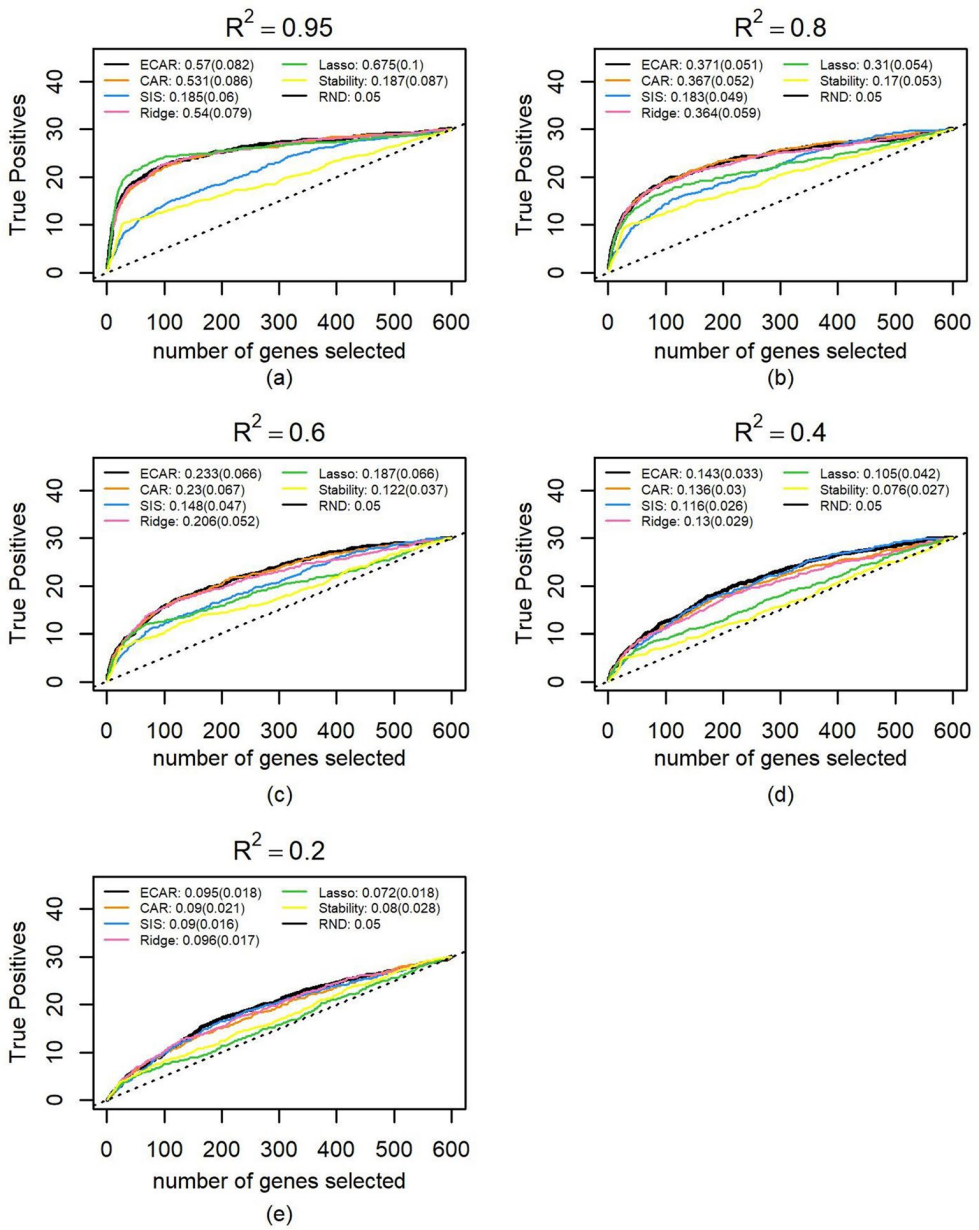
Comparison of feature selection performance on 500 simulated datasets. This figure is the same as Fig. 3 except that the influential features are selected from the whole set of features randomly.

Since it seems too advantageous to SIS that the influential genes are all from the first block, we also tried to select the genes from the whole set randomly. Figure 5 shows that ECAR outperforms SIS, ridge, and stability selection consistently and is highly competitive to lasso except when noise is extremely low ($R^{2}$ = 0.95). The path of ECAR and CAR is very similar in the figure, which indicates the result is not very sensitive to $\alpha$ in this study. We also performed the sensitivity analysis in which different coefficient distributions are used to estimate $\alpha$. We noticed that the result of ECAR may be affected when the coefficients of features in the test sets are generated from an entirely different distribution from the one (uniform distribution) we use in estimating $\alpha$, the result may change a bit. For example, when the coefficients of features in the test sets are sampled from the standard normal distribution, the performance of lasso and stability selection is greatly enhanced. At the same time, ECAR, CAR, and ridge select fewer true positives. This decline of performance is due to their grouping property (positively correlated features tend to have the same scores). If the positively correlated influential features’ coefficients are sampled from the standard normal distribution and thus have different signs, these features’ scores would tend to cancel each other out, and this will undermine the performance of these methods. However, the effect is not significant, as can be seen in Supplementary Table S2.

### Comparison of variable selection accuracy on real mRNA expression datasets with simulated phenotypes

**Figure 6:**
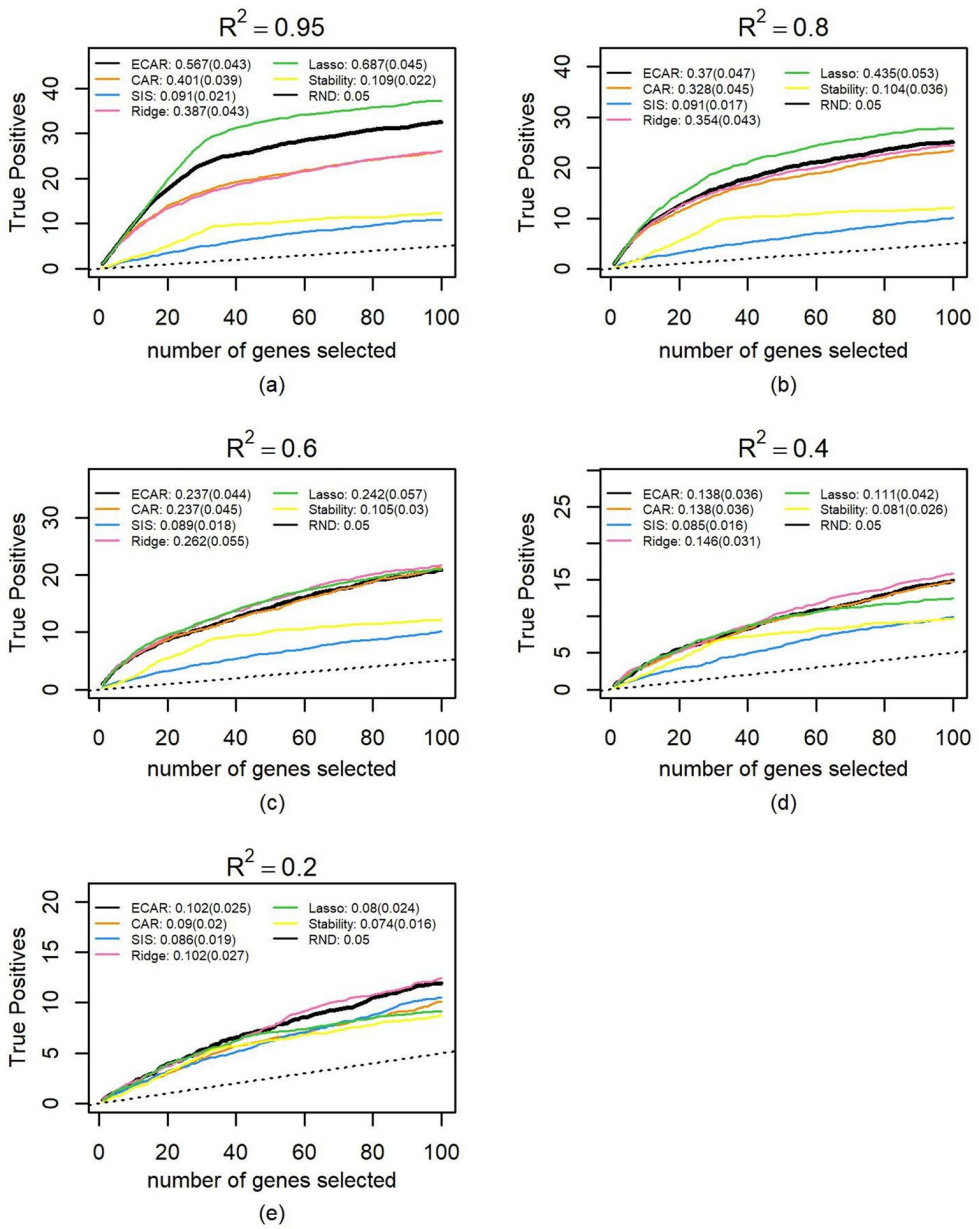
Comparison of variable selection performance on 500 semi-synthetic datasets. The median number of true positive variables as a function of the total number of selected genes as well as the median of PR-AUC and its standard deviation are shown for ECAR, CAR, SIS, ridge, lasso and stability selection under five $R^{2}$ scenarios. The total number of influential genes is 50, which are randomly selected from the 1000 genes. Parameter ${\upalpha }$ of ECAR is estimated using the methods described in the Methods section. The regularization parameter of ridge and lasso is estimated using fivefold cross-validation and generalized cross-validation, respectively. As lasso cannot select more variables than the sample size, we let it choose genes randomly when all genes in the output selected set are chosen. (**a**) $R^{2}$ is controlled at 0.95 for the 100 simulated datasets. (**b**) Same as (**a**), $R^{2}$ controlled at 0.8. (**c**) Same as (**a**), $R^{2}$ controlled at 0.6. (**d**) Same as (**a**), $R^{2}$ controlled at 0.4. (**e**) Same as (**a**), $R^{2}$ controlled at 0.2.

We obtained mRNA expression datasets from 3 cancer projects (LUAD, LIHC, LUSC) from The Cancer Genome Atlas (TCGA) portal (https://tcga-data.nci.nih.gov/tcga/) using TCGA-Assembler^27^ (v1.0.3). We removed the genes with zero expression in more than 20% of the samples. For each expression data, 1000 genes with the largest variance were selected, log-transformed and normalized to form the simulation dataset. The sample sizes of simulation datasets are 512, 369, and 497, respectively. The comparison method is the same as that in the previous section. Figure 6 summarizes the performance of feature selection for each method on the LUAD dataset (results on LIHC and LUSC are similar). The medians of the best ${\upalpha }$ and corresponding standard deviations when $R^{2}$ = 0.2, 0.4, 0.6, 0.8 and 0.95 are 0.3 ± 0.13, 0.45 ± 0.07, 0.55 ± 0.05, 0.65 ± 0.05 and 0.80 ± 0.06, respectively. Figure 6 looks similar to Fig. 3, as ECAR still outperforms CAR when $R^{2}$ is above 0.6 or below 0.4, and it selects more true positives than SIS and stability selection consistently. Compared with the more artificial examples in the previous section, lasso performs better as its advantage over ECAR maintains until $R^{2}$ drops to 0.6. When there is high noise ($R^{2}$ = 0.2), all methods behave similarly. These results further demonstrate that when the features have a general correlation structure, ECAR still improves CAR, and is competitive to some classic variable selection methods. We also performed several sensitivity analyses, in which we changed the distribution of coefficient, number of influential genes. It turns out the results are quite similar to those shown here (Supplementary Fig. S6–S8). Another interesting thing is that the best $\alpha$ seems to be insensitive to the dimension: when we increased the dimension to 4000, the best $\alpha$ under different $R^{2}$ levels remains almost the same.

### ECAR applied to barley dataset

The data we used were downloaded from the T3/barley database. The sequencing data are from a genotyping experiment, BarleyNB_9K (Platform: Infinium 9K). Barley samples have spike length information in the trial of experiment NSGC Spring Core Panel in 2012, and lodging degree and leaf width information in the trail of experiment UMN NSGC GWAS in 2015. Genotyping experiment BarleyNB_9K includes 2417 samples and 6913 markers. We cleaned this data by removing 63 markers with a minor allele frequency (MAF) less than 1% and 429 markers which are missing for more than 3% of data. In the data, 1, 0, − 1 represent AA, AB, BB respectively. The missing values were replaced by − 1. After removing samples with NA trait value and markers that are duplicated or have zero variance, the spike length dataset contains information for 1947 samples and 6583 markers; the lodging degree dataset has 712 samples and 6236 markers; the leaf width dataset has 738 samples and 6239 markers.

We applied ECAR, CAR, lasso, stability selection, SIS, ridge on these three datasets. Since we do not know the truly influential SNPs, we compared the mean square error (MSE) on the test set indexed by the number of SNPs in the model instead. For the spike length, lodging degree, and leaf width dataset, the $R^{2}$ are estimated to be 0.4, 0.32, and 0.45; the numbers of influential variables are estimated to be 326, 152, and 145; parameter $\alpha$ is estimated to be 0.45 ± 0.03, 0.4 ± 0.07, and 0.5 ± 0.06.

Tables 1 and 2 summarizes the generalization performance of high-rank SNP features evaluated by lasso and ridge on the three datasets. The generalization performance is assessed by the MSE of lasso or ridge on the test sets. From the two tables, we can see that while none of these methods perform consistently well in every case, SNPs ranked by ECAR scores have relatively high prediction accuracy overall. The MSE of ECAR is less than CAR, lasso, and ridge in all cases except in the leaf width dataset where ECAR is equivalent to CAR $\left( {\alpha = 0.5} \right)$. Stability selection seems to perform quite well in terms of prediction error among multivariate methods. We noticed that in the lodging degree and the leaf width dataset, SIS has superior MSE over other methods. However, we showed that it has relatively lower feature selection accuracy compared to other methods in the previous sections. This phenomenon demonstrates that a feature with high predictive power does not necessarily have a causal relationship with the outcome. To sum up, these three examples show that even though ECAR is designed explicitly for selecting predictive variables, it is competitive to other classic multivariate methods in terms of predictive power.

**Table 1:** Summary of the generalization performance of high-rank SNP features evaluated by lasso on the three datasets.

Data	Features’ number	ECAR	CAR	Lasso	Stability selection	SIS	Ridge	Base lasso
Spike Length (${R}^{2}\hspace{0.17em}$= 0.4, $\mathrm{\alpha }\hspace{0.17em}\hspace{0.17em}$= 0.45)	5	342.9	357.3	361.1	331.4	331.2	366.1	272.8
	10	320.4	330.1	339.0	317.1	325.0	332.2	272.8
	20	303.6	308.7	314.9	301.1	317.8	317.0	272.8
	30	301.9	306.2	310.2	296.3	316.1	307.2	272.8
Lodging Degree (${R}^{2}\hspace{0.17em}$= 0.32, $\mathrm{\alpha }\hspace{0.17em}$= 0.4)	5	3.48	3.56	3.57	3.51	3.15	3.80	2.91
	10	3.42	3.54	3.49	3.35	3.10	3.75	2.91
	20	3.36	3.39	3.46	3.30	3.14	3.60	2.91
	30	3.28	3.43	3.47	3.27	3.12	3.52	2.91
Leaf Width (${R}^{2}\hspace{0.17em}$= 0.45 $\mathrm{\alpha }\hspace{0.17em}$= 0.5)	5	0.067	0.067	0.070	0.062	0.060	0.069	0.050
	10	0.061	0.061	0.063	0.059	0.056	0.066	0.050
	20	0.058	0.058	0.059	0.056	0.055	0.062	0.050
	30	0.056	0.056	0.059	0.055	0.059	0.060	0.050

Base lasso is the prediction performance of lasso on the test sets using all features as input. See the “Methods” section for further details.

**Table 2:** Summary of the generalization performance of high-rank SNP features evaluated by ridge on the three datasets.

Data	Features’ number	ECAR	CAR	Lasso	Stability selection	SIS	Ridge	Base ridge
Spike Length (${R}^{2}\hspace{0.17em}$= 0.4, $\mathrm{\alpha }\hspace{0.17em}$= 0.45)	5	344.5	350.5	361.6	336.6	337.8	350.1	268.3
	10	326.5	329.4	340.4	319.7	331.8	331.4	268.3
	20	303.8	305.7	317.9	300.9	324.6	310.1	268.3
	30	297.7	301.4	305.5	297.2	324.8	303.1	268.3
Lodging Degree (${R}^{2}\hspace{0.17em}$= 0.32, $\mathrm{\alpha }\hspace{0.17em}$= 0.4)	5	3.45	3.46	3.44	3.34	3.10	3.69	2.61
	10	3.30	3.46	3.42	3.24	3.00	3.63	2.61
	20	3.26	3.39	3.33	3.18	2.95	3.56	2.61
	30	3.21	3.29	3.36	3.18	2.91	3.40	2.61
Leaf Width (${R}^{2}\hspace{0.17em}$= 0.45, $\mathrm{\alpha }\hspace{0.17em}$= 0.5)	5	0.065	0.065	0.066	0.062	0.058	0.070	0.047
	10	0.061	0.061	0.061	0.057	0.055	0.067	0.047
	20	0.057	0.057	0.059	0.054	0.053	0.062	0.047
	30	0.055	0.055	0.058	0.053	0.052	0.060	0.047

Base ridge is the prediction performance of ridge regression on the test sets using all features as input. See the “Methods” section for further details.

### ECAR applied to the LUAD dataset

We analyzed forced expired volume in 1 s (FEV1) in 230 patients from the TCGA LUAD cohort (total n = 577). In the data preprocessing step, 3781 genes which have zero value for more than 20% of samples were removed. This results in a dataset of 230 samples and 16,750 genes. After the logarithmic transformation on both the gene expressions and FEV1, we performed ECAR on the data.

The ${R}^{2}$ and the number of influential variables were estimated to be 0.1 and 9, respectively. In this data, $\alpha$ estimates had a median of 0.15 and a standard deviation of 0.28. Controlling the false discovery rate at 5%, ECAR selected six genes *CHRM3, CTCFL, KCNE2, MLANA, MSMP, TTLL2*, many of which have been reported to be associated with lung function or cancer. For example, *CHRM3* encodes the muscarinic acetylcholine receptor M3, which is a well-characterized drug target for which many approved drugs exist, including for the treatment of asthma and obstructive lung disease^28^. *BORIS* transcripts were expressed in lung carcinoma cell lines at high to moderate levels^29^. *KCNE2* and *TTLL2* might be associated with pulmonary function^30,31^. *MSMP* hindered the effect of anti-VEGF therapy^32^, and it could promote xenograft PC3 growth and reduce the survival of PC3 metastatic mice model^33^. The genes selected by SIS and CAR are listed in Table 3.

**Table 3:** Summary of selected genes for each method.

Methods	Selected genes (FDR = 0.05)
ECAR	*CHRM3, CTCFL, KCNE2, MLANA, MSMP, TTLL2*
CAR	*A4GNT, ALPL, ANKRD55, C3orf32, C5orf38, C6orf138, C9orf70, CEACAM7, CHRM3, COL11A2, CTCFL, GJC3, GRIN2A, GSTT2, KCNE2* *LOC440461, MLANA, MSMP, MUC6, MYOT, NUDT12, POMZP3, PRR4, SRCRB4D, TRIM61, TTLL2*
SIS	*MSMP*

## Discussion

We developed the ECAR scores, which can simultaneously measure the importance of all the variables in regression models. We showed that by diligently searching the parameter which maximizes PR-AUC, ECAR can improve traditional methods like SIS, ridge, CAR in terms of the feature selection accuracy, while maintaining strong predictive power in high-rank features. ECAR is also highly competitive to popular variable selection methods like lasso, notably when influential factors are correlated. Moreover, it enjoys the grouping property that strongly correlated variables will tend to be selected together. Another advantage of ECAR is that its parameter is insensitive to the sampling setting: even the coefficients’ distribution used is different from the real one, the results would not be significantly different. The flexibility of ECAR not only enables it to perform well under settings that are unsuitable for CAR, but also ensure it has approximately equal performance when CAR works satisfactorily. Some researchers applied the CAR scores to SNP selection in the GAW17 dataset and found CAR performed much better than other classic methods^34^. We tried to estimate $\alpha$ in this dataset, and it turned out the best $\alpha$ is very close to 0.5, which explains why CAR can perform better than those classic methods in this data.

One issue with ECAR is that it is computationally intensive when estimating the parameter $\alpha$, since we have to compute the power of the correlation matrix 21 times. This process can be accelerated using the methods proposed by Strimmer^21^, which enables substantial computational savings when the sample size is much smaller than the number of features. Another issue is that it is not a completely automatic method, which means we have to estimate some parameters like ${R}^{2}$ and the number of influential variables. The estimation accuracy will affect the result, but luckily the effect is limited. Finally, when the data has a small ${R}^{2}$ or number of influential variables, the standard deviation of $\alpha$ may be quite large, and it might be more secure to apply the more conservative SIS in this case.

## Methods

### Data

To evaluate the performance of the ECAR scores, we used three kinds of datasets. The first kind of datasets has 200 samples and 600 features which were split into two equal-sized groups. The datasets were generated from a multivariate normal distribution with mean zero. The correlations between any two features in the same group are 0.25, and features in different groups are independent. The outcomes were generated by a Gaussian linear model in which influential features are randomly selected. The second datasets we used were semi-synthetic datasets. We called it semi-synthetic datasets because the features are from real mRNA expression data obtained from 3 cancer projects (LUAD, LIHC, LUSC) from TCGA. The outcomes were generated in the same way as described above. We compared the variable selection accuracy on the datasets mentioned above. We also used three barley datasets downloaded from the T3/barley database whose sample and feature size ranged between 712 to 1947 and 6236 to 6583, to evaluate the prediction performance of ECAR. We applied ECAR to the LUAD dataset and analyzed genes that influence forced expired volume in 1 s (FEV1). The information of datasets is shown in Supplementary Table S1.

### Details of the ECAR scores

Our method takes genomic features and the outcome as input and returns scores that represent the importance of features. In ECAR scores ${{\varvec{R}}}^{-\alpha }{{\varvec{R}}}_{{\varvec{X}}Y}$ (Eq. 1), the calculation of Pearson Correlation Coefficient ${{\varvec{R}}}_{{\varvec{X}}Y}$ is straightforward. As for ${{\varvec{R}}}^{-\alpha }$, we need to estimate $\alpha$ from data, and then we can use function powcor.shrink from R package corpcor to calculate ${{\varvec{R}}}^{-\alpha }$. The procedures for estimating $\alpha$ is as follows. First, to simplify the computation, we limit the choice of $\alpha$ to an equally spaced sequence which contains 21 numbers ranging between 0 and 1 (0, 0.05, 0.1, …,1). Second, we simulate 100 datasets using a Gaussian linear model ${\varvec{Y}}={\varvec{X}}{\varvec{\beta}}+{\varvec{\varepsilon}}$, where ${\varvec{\varepsilon}}\sim {N}_{n}(0,{\sigma }^{2}{{\varvec{I}}}_{n})$. In this model, ${\varvec{X}}$ is given and we need to predetermine ${\varvec{\beta}}$ and $\sigma^{2}$ to generate ${\varvec{Y}}$. If $R^{2}$ and the number of influential variables $s$ are already known approximately to us, we can just randomly select $s$ variables whose corresponding ${\varvec{\beta}}$ are sampled from the uniform distribution with minimum 0 and maximum 1 (the rest of the ${\varvec{\beta}}$ are 0); the $\sigma^{2}$ is set to be $\frac{{\left( {1 - R^{2} } \right){\varvec{\beta}}^{{\varvec{T}}} {\varvec{X}}^{{\varvec{T}}} \user2{X\beta }}}{{nR^{2} }}$ so that the model can achieve the predetermined $R^{2}$. If $R^{2}$ and $s$ are not given, we can estimate them from the data. Many methods can be applied to estimate $R^{2}$, and refitted cross-validation^35^ is an example. The number of influential features $s$ can be estimated based on the cardinality of lasso’s selected set, and the regularization parameter of lasso can be estimated by generalized cross-validation to avoid numerical instability. Our sensitivity analysis results showed that the result is not very sensitive to these estimates. To ensure that ${\varvec{R}}$ is positive definite, we use the shrinkage approach proposed by Strimmer et al.^36^ implemented in R package corpcor. After generating the simulated datasets, the PR-AUC (Prediction-Recall Area Under Curve) can be computed at each value of $\alpha$, and for each dataset the $\alpha$ that maximizes PR-AUC will be selected. We take the median of these $\alpha$ values to be the estimate for $\alpha$. To work out a cutout for the scores and achieves false discovery control, we apply the adaptive false discovery rate density approach^23^ (using function fdrtool from R package fdrtool).

### Evaluation measures

The performance of ECAR was evaluated in terms of feature selection and prediction accuracy. ECAR assigns a score to each feature in the dataset, and these features are later ranked and selected by the model in descending order according to the absolute value of their scores. For the evaluation of ECAR’s performance on simulated datasets whose true influential features are known, we used PR-AUC, which is the area under the precision-recall curve created by plotting the precision against the recall at various threshold settings. The precision is the number of true positive factors divided by the number of selected factors, while recall is the fraction of true positive factors that are retrieved. We also plotted the number of true positive factors against the number of total selected factors. For the real data, we looked at the prediction performance of the selected SNP features. The whole data were randomly split into a training set of 2/3 of total samples and a test set of the remaining 1/3. We reported the test performance evaluated by MSE (mean square error) averaged across ten different random divisions of training and test sets. For all the methods, we showed the mean of MSE for the number of selected features $s$ from the set (5, 10, 20, 30).

### Sensitivity analysis

To test the stability of $\alpha$ under different sampling settings, we perform the sensitivity analysis in which three distributions (uniform, normal and folded normal) of coefficients are used as the real distribution, and in all cases we only use uniform distribution in estimating $\alpha$. For the datasets mentioned above, we studied the influence of the misspecification of parameters to the feature selection performance (Supplementary Fig. S1–S8 and Supplementary Table S2).

### Comparison with other methods

ECAR was compared with CAR, SIS, Ridge, Lasso, stability selection and random selection (randomly rank the features, denoted by RND) in terms of feature selection accuracy and generalization performance of high-rank features. The rank of the variables is based on their scores calculated from different methods. Sure Independence Screening (SIS) is a univariate correlation ranking method that ranks features’ importance according to their marginal correlation with the response variable. It is helpful in ultra-high dimension settings for screening irrelevant variables; however, it could lead to a high rate of false positives regarding importance ranking problems in moderately high dimension settings. In our study, the scores are the absolute value of Pearson Correlation Coefficient ${\varvec{R}}_{{{\varvec{X}}Y}}$. Lasso and Ridge are specific cases of bridge estimators $\hat{\user2{\beta }} = \arg \mathop {\min }\limits_{{{\varvec{\beta}} \in {\varvec{R}}^{{\varvec{p}}} }} \frac{1}{n}||{\varvec{y}} - \user2{X\beta }\left| {|^{2} + \lambda } \right|\left| {\varvec{\beta}} \right||_{q}^{q}$ when ${\text{q}} = 1$ and ${\text{q}} = 2$, where ${\varvec{X}}$ is ${\text{n}} \times {\text{p}}$ design matrix, and ${\mathbf{y}}$ is ${\text{n}} \times 1$ response vector. When used for feature ranking, a two-stage variable selection (TVS) technique will be applied. The first stage computes the bridge estimators, and the second stage thresholds this estimate to rank the predictors. A critical difference between these two methods is that Lasso gives a set of zero regression coefficients and leads to a sparse solution. A well-known problem with Lasso is that it tends to select only one variable from a group of highly correlated genomic factors. Also, it cannot select more variables than the sample size. For Ridge and Lasso in this study, their parameter $\lambda$ are estimated by fivefold cross-validation and generalized cross-validation. Stability selection ranks each variable by the probability of being selected by specific selection methods such as Lasso. It is better suited for variable selection, but the price to pay is the reduced power to identify true signals. In stability selection, we first draw 100 subsamples of size ${\text{n}}/2$ without replacement, and then apply lasso on these subsamples and our scores are the frequency of each feature being selected. As for choosing the parameters, we set the number of false positives $v$ to be 2.5 and the cutout $\pi_{{{\text{cut}}}}$ to be 0.7; therefore, the regularization parameter should be adjusted to ensure $\sqrt {vp\left( {2\pi_{{{\text{cut}}}} - 1} \right)}$ ($p$ is the total number of variables) features are selected in the model in each replicate, according to the result in the paper^20^. Another approach used for feature ranking is CAR scores, which are calculated based on the correlations between the de-correlated variables and the response variable. The calculation of CAR scores is relatively simple, which is ${\varvec{R}}^{ - 0.5} {\varvec{R}}_{{{\varvec{X}}Y}}$, yet it performs well in some cases. However it could be too aggressive or conservative in real problems.

### Software

R (v.3.6.0) was used for the development of ECAR; pROC (v.1.15.0) was used for computing PR-AUC; corpcor (v.1.6.9) was used for calculating the α power of the correlation matrix. glmnet (v.3.0-2) was used for comparison with ECAR. mvtnorm (v.1.0-10) was used for generating random numbers for the multivariate normal distribution.

## Supplementary Information

Supplementary Information.

## Data Availability

The study used multiple publicly available datasets. The data for three cancer projects LUAD, LUSC and LIHC are obtained from The Cancer Genome Atlas (TCGA) portal (https://tcga-data.nci.nih.gov/tcga/). Three GWAS datasets are obtained from T3/barley database (https://triticeaetoolbox.org/barley/).
