# Supplementary material for: Elastic Correlation Adjusted Regression (ECAR) scores for high dimensional variable importance measuring
Source: Sci Rep. 2021 Dec 2;11:23354. doi: 10.1038/s41598-021-02706-0 (PMC8640025; doi:10.1038/s41598-021-02706-0)
Supplement: Supplementary file 1 — Supplementary Information. [file 41598_2021_2706_MOESM1_ESM.pdf]

Elastic Correlation Adjusted Regression (ECAR) Scores for High Dimensional Variable Importance Measuring

Yuan Zhou[1], Botao Fa[2], Ting Wei[2], Jianle Sun[2], Zhangsheng Yu[2]*,Yue Zhang[2]*

[1]Department of Mathematical Sciences, University of Cincinnati, Cincinnati, Ohio, USA
[2]School of Life Sciences and Biotechnology, Shanghai Jiao Tong University, Shanghai, China
*email:
Zhangsheng Yu: yuzhangsheng@sjtu.edu.cn
Yue Zhang: yue.zhang@sjtu.edu.cn

Supplementary Information:

Feature Type	Source	Sample Size	Feature Size
Simulated	-	200	600
mRNA Expression (simulated outcome)	1. LUAD (TCGA)	512	1000
	2. LUSC (TCGA)	497	1000
	3. LIHC (TCGA)	369	1000
SNP	1. Spike Length (T3/barley)	1947	6583
	2. Lodging Degree (T3/barley)	712	6236
	3. Leaf Width (T3/barley)	738	6239
mRNA Expression (FEV1)	1. LUAD (TCGA)	230	16750

Supplementary Table 1. Datasets used for the ECAR

Datasets	True Distribution	Used Distribution	R^2	PR-AUC1	PR-AUC2
simulated	uniform	uniform	0.95	0.563(0.063)	0.553(0.049)
			0.8	0.354(0.085)	0.369(0.047)
			0.6	0.216(0.051)	0.22(0.049)
			0.4	0.126(0.033)	0.133(0.043)
			0.2	0.1(0.033)	0.092(0.023)
simulated	normal	uniform	0.95	0.469(0.064)	0.448(0.057)
			0.8	0.398(0.064)	0.372(0.063)
			0.6	0.289(0.056)	0.27(0.075)
			0.4	0.196(0.053)	0.185(0.052)
			0.2	0.109(0.041)	0.118(0.049)
simulated	folded normal	uniform	0.95	0.477(0.078)	0.497(0.065)

			0.8	0.364(0.05)	0.346(0.045)
			0.6	0.217(0.046)	0.218(0.062)
			0.4	0.136(0.034)	0.129(0.032)
			0.2	0.095(0.021)	0.082(0.027)
semi-synthetic	uniform	uniform	0.95	0.57(0.052)	0.55(0.056)
			0.8	0.368(0.062)	0.347(0.065)
			0.6	0.222(0.051)	0.234(0.052)
			0.4	0.152(0.039)	0.152(0.033)
			0.2	0.094(0.023)	0.099(0.023)
semi-synthetic	normal	uniform	0.95	0.485(0.06)	0.489(0.048)
			0.8	0.376(0.038)	0.378(0.051)
			0.6	0.273(0.049)	0.238(0.038)
			0.4	0.164(0.032)	0.155(0.037)
			0.2	0.096(0.02)	0.1(0.022)
semi-synthetic	folded normal	uniform	0.95	0.494(0.062)	0.498(0.05)
			0.8	0.328(0.046)	0.346(0.05)
			0.6	0.222(0.041)	0.235(0.055)
			0.4	0.154(0.032)	0.138(0.044)
			0.2	0.094(0.023)	0.096(0.029)

Supplementary Table 2. The sensitivity of results to the misspecification of parameters
To compute PR-AUC1, we use the true coefficients' distribution, R^2, and s when estimating α. As for PR-AUC2, we use the uniform distribution, estimated R^2 and s.

Sensitivity Analysis:

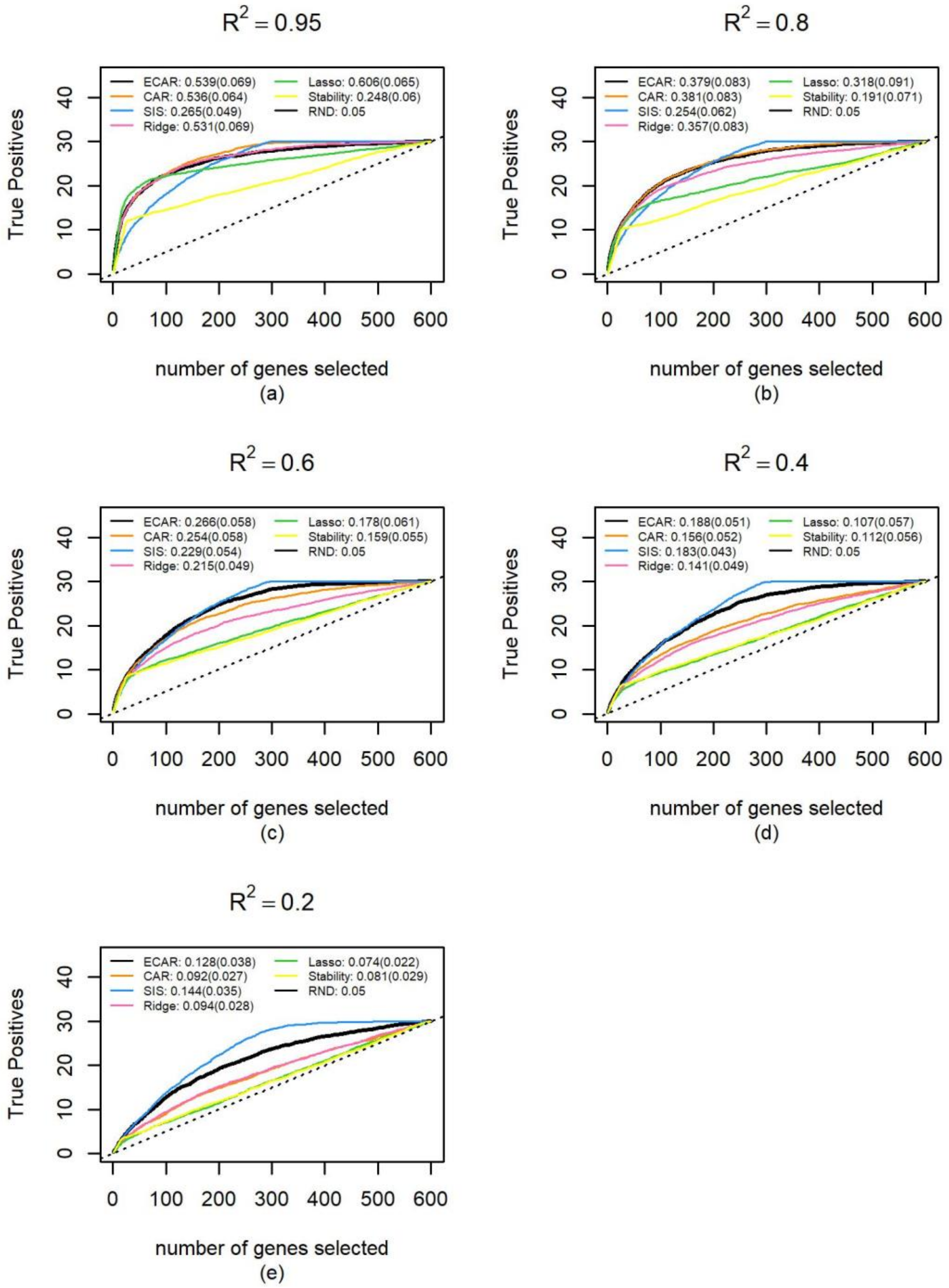

Supplementary Figure 1. Influence of misspecification of parameters.

For the simulated test datasets, influential features are selected from the first block (first 300) and their coefficients in the model are sampled from the folded standard normal distribution. In α estimation procedure, coefficients' distributions are uniform. R^2 and s are estimated by the refitted cross-validation and lasso, respectively.

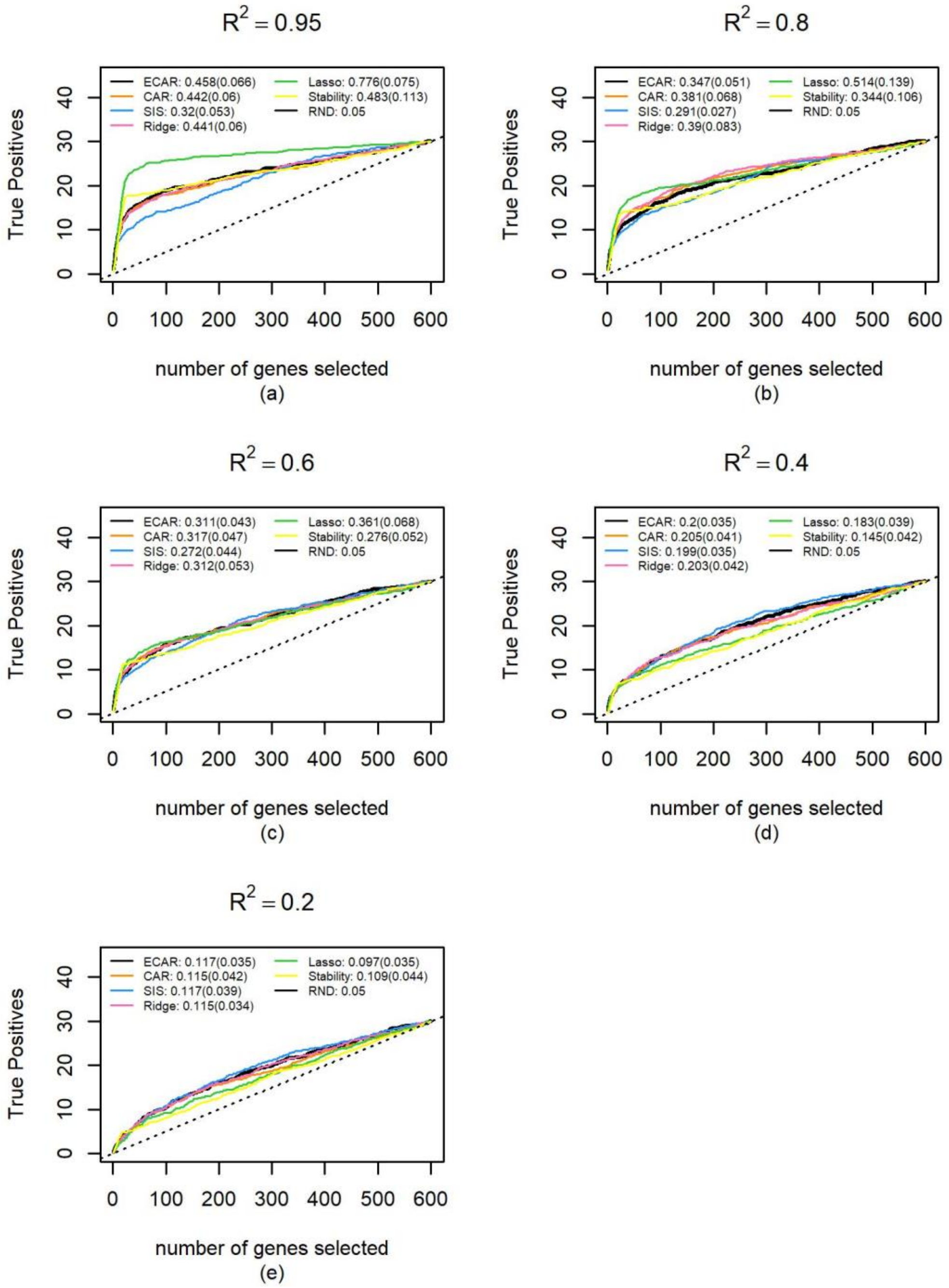

Supplementary Figure 2. Influence of misspecification of parameters.

For the simulated test datasets, 30 influential features are selected from the first block (first 300), and their coefficients in the model are sampled from the standard normal distribution. In α estimation procedure, coefficients' distributions are uniform. R^2 and s are estimated by the refitted cross-validation and lasso, respectively.

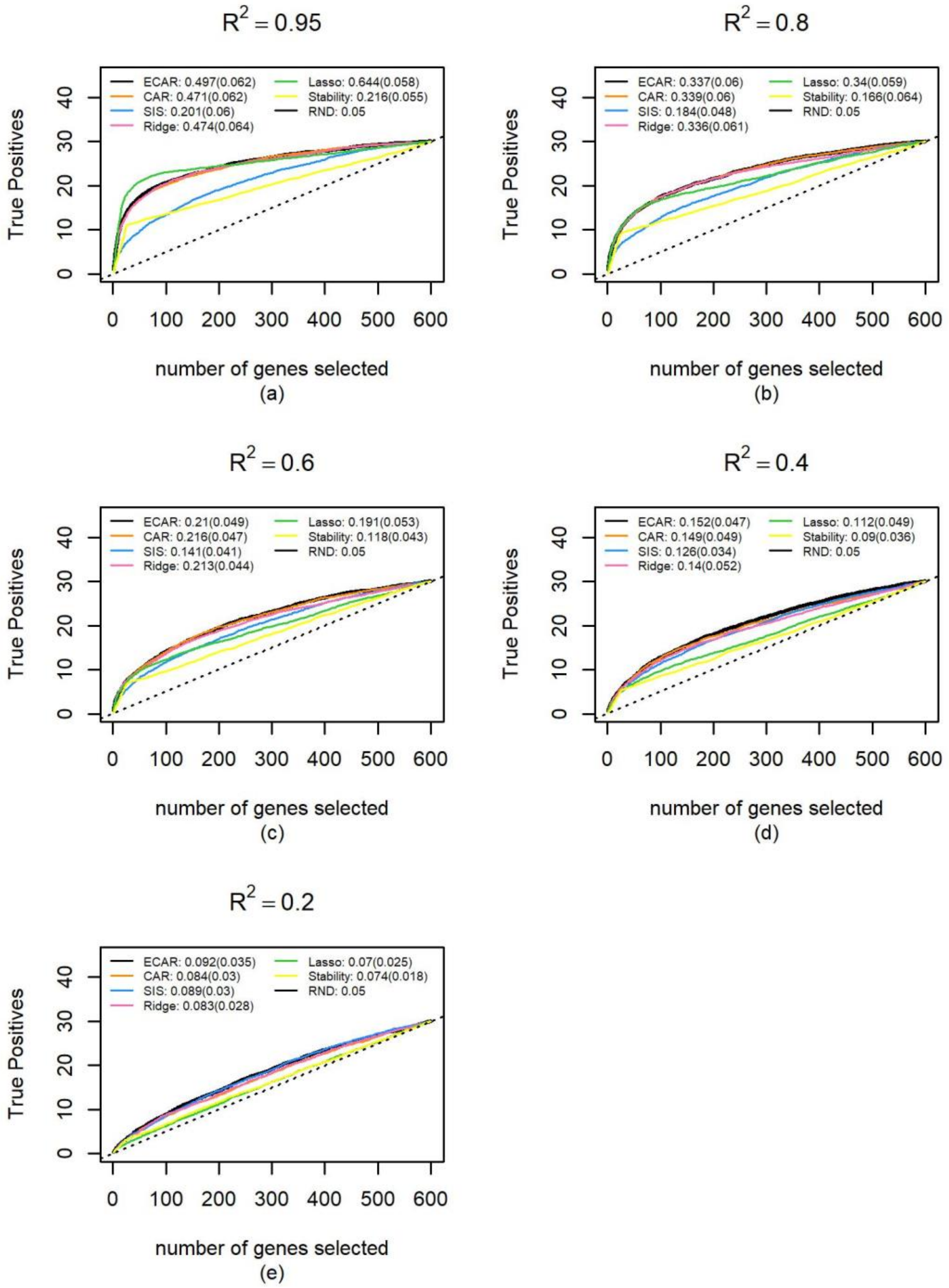

Supplementary Figure 3. Influence of misspecification of parameters.

For the simulated test datasets, 30 influential features are selected from the whole set of variables, and their coefficients in the model are sampled from the folded standard normal distribution. In α estimation procedure, coefficients' distributions are uniform. R^2 and s are estimated by the refitted cross-validation and lasso, respectively.

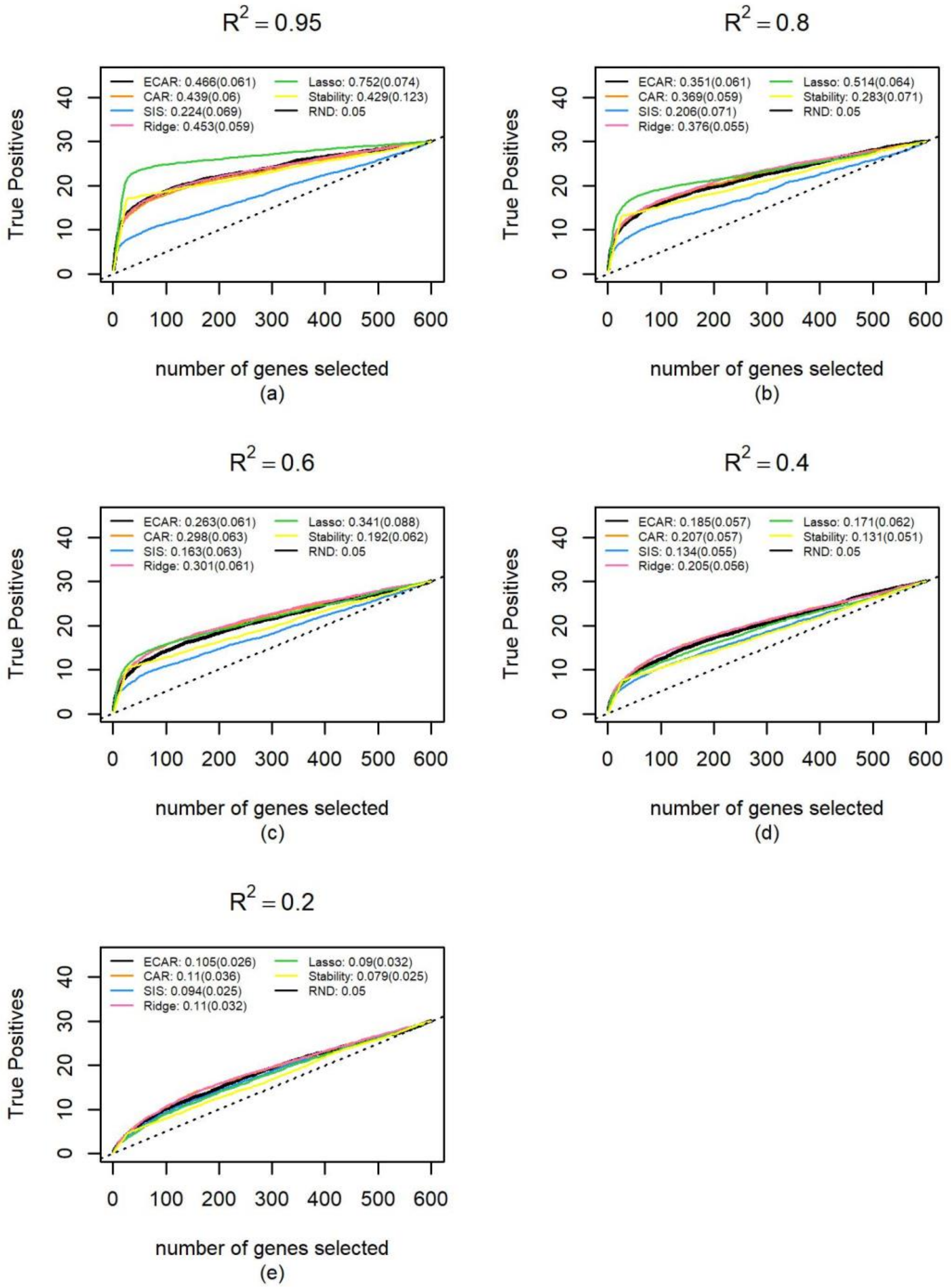

Supplementary Figure 4. Influence of misspecification of parameters.

For the simulated test datasets, 30 influential features are selected from the whole set of variables, and their coefficients in the model are sampled from the standard normal distribution. In α estimation procedure, coefficients' distributions are uniform. R^2 and s are estimated by the refitted cross-validation and lasso, respectively.

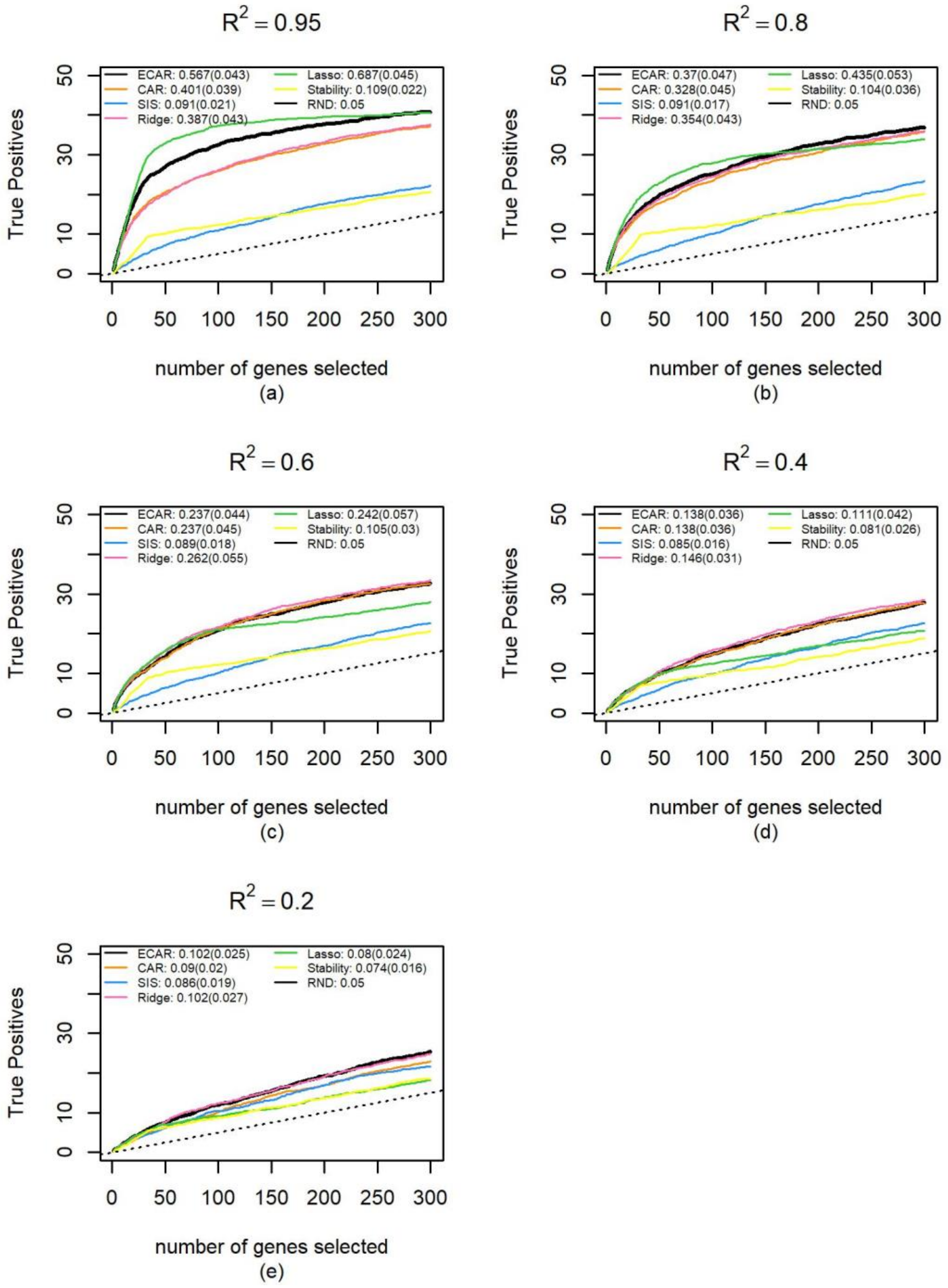

Supplementary Figure 5. Influence of misspecification of parameters.

For the semi-synthetic datasets, 50 influential features are selected from the whole set of variables, and their coefficients in the model are sampled from uniform distribution. In α estimation procedure, coefficients' distributions are uniform. R^2 and s are not estimated here, and therefore they equal to their true values. The paths are truncated at 300 genes.

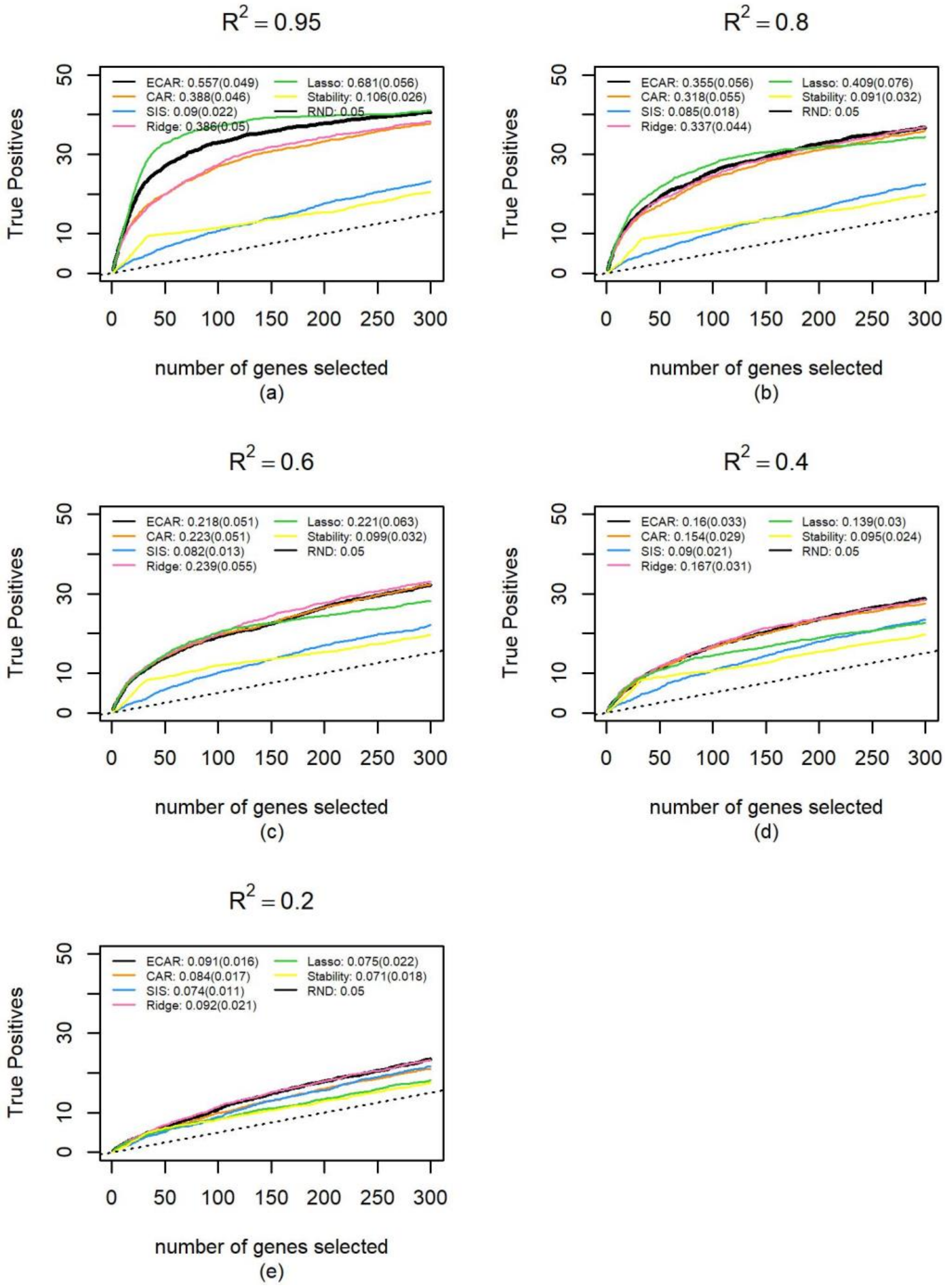

Supplementary Figure 6. Influence of misspecification of parameters.

For the semi-synthetic datasets, 50 influential features are selected from the whole set of variables and their coefficients in the model are sampled from the uniform distribution. In α estimation procedure, coefficients' distributions are uniform. R^2 and s are estimated by the refitted cross-validation and lasso, respectively. The paths are truncated at 300 genes.

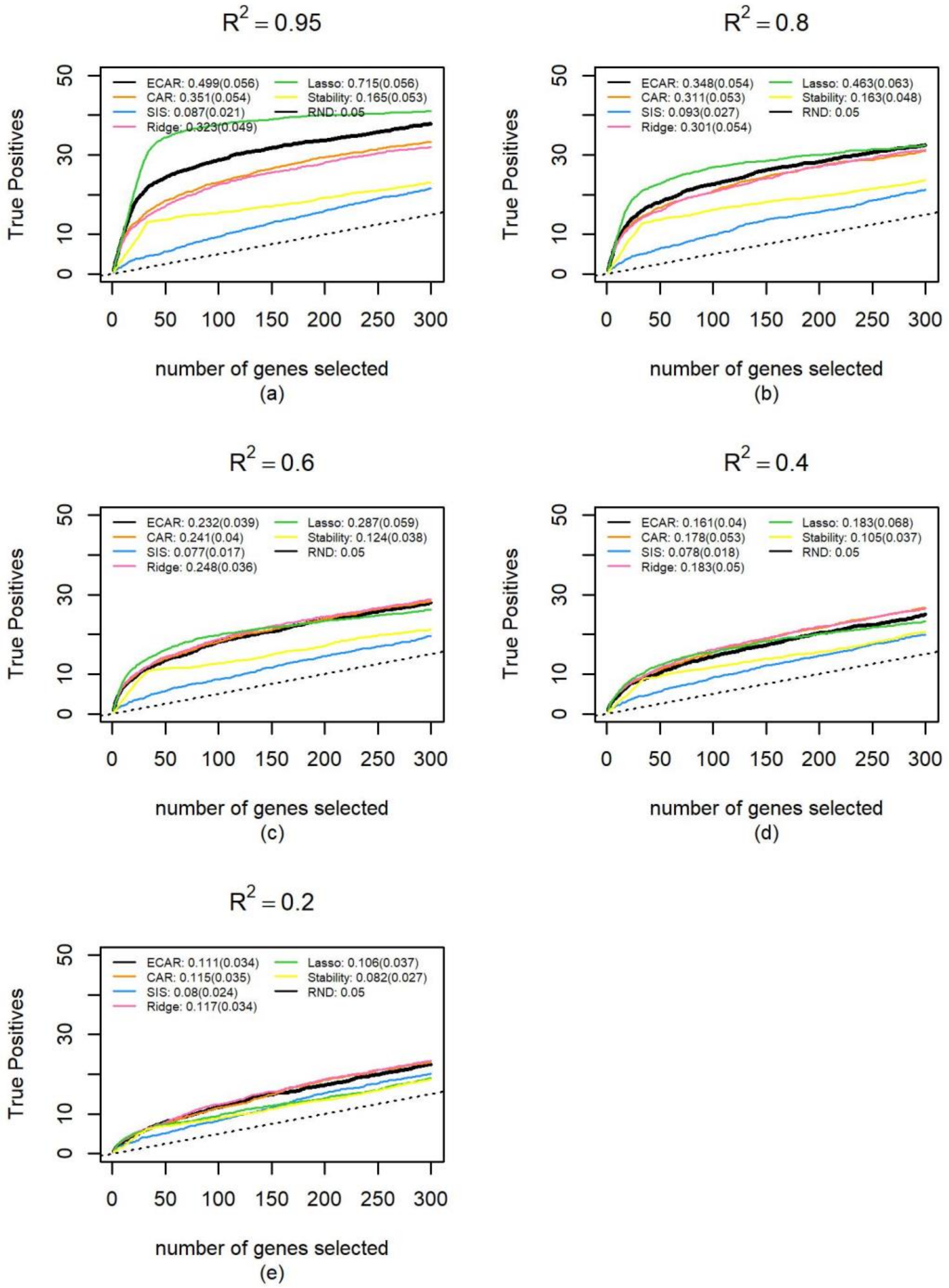

Supplementary Figure 7. Influence of misspecification of parameters.

For the semi-synthetic datasets, 50 influential features are selected from the whole set of variables, and their coefficients in the model are sampled from the standard normal distribution. In α estimation procedure, coefficients' distributions are uniform. R^2 and s are estimated by the refitted cross-validation and lasso, respectively. The paths are truncated at 300 genes.

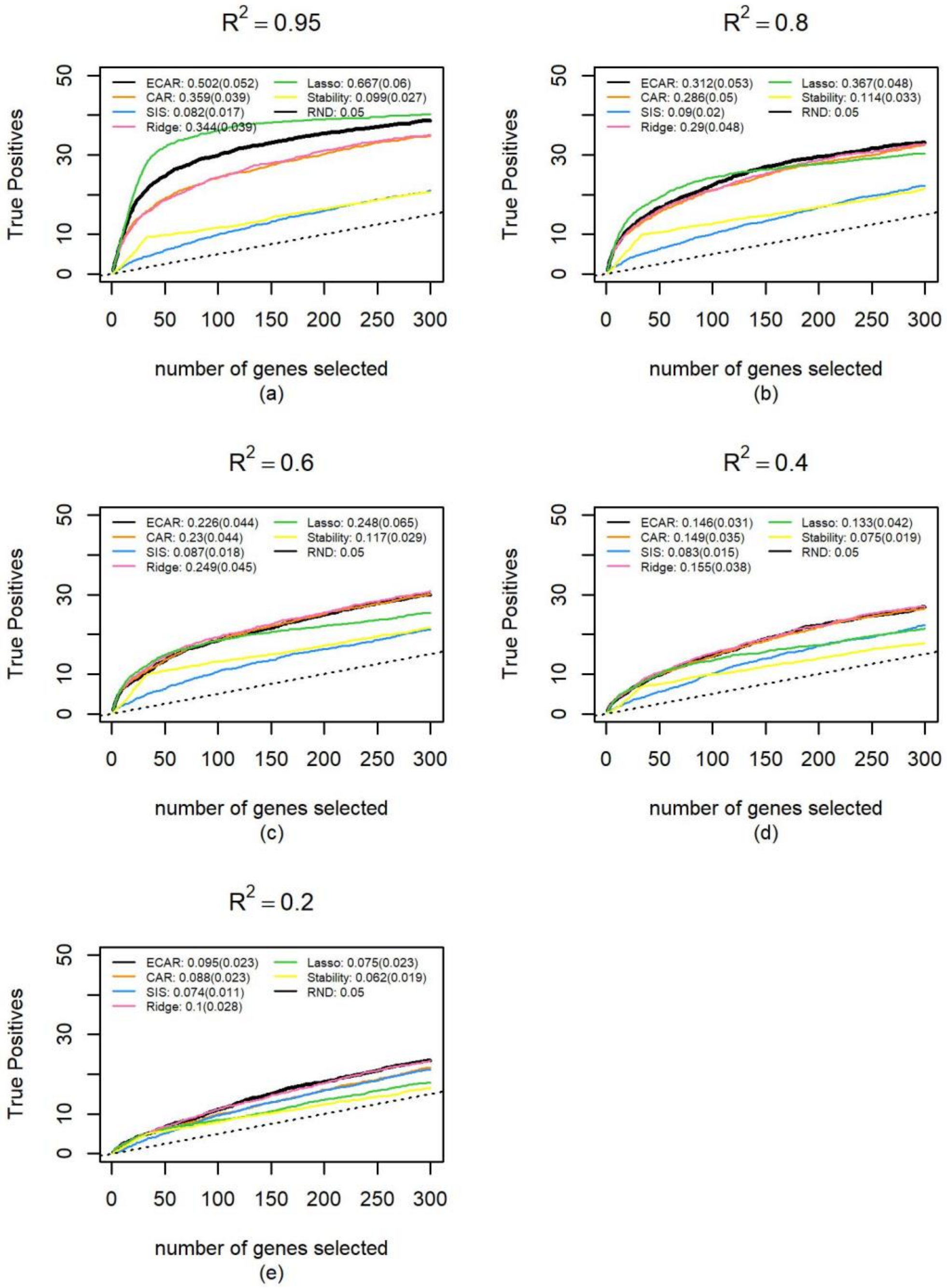

Supplementary Figure 8. Influence of misspecification of parameters.

For the semi-synthetic datasets, 50 influential features are selected from the whole set of variables, and their coefficients in the model are sampled from the folded normal distribution. In α estimation procedure, coefficients' distributions are uniform. R^2 and s are estimated by the refitted cross-validation and lasso, respectively. The paths are truncated at 300 genes.
